# Pan-cancer analysis of the prognosis and immunological role of AKAP12: A potential biomarker for resistance to anti-VEGF inhibitors

**DOI:** 10.3389/fgene.2022.943006

**Published:** 2022-08-30

**Authors:** Qiuju Liang, Jinwu Peng, Zhijie Xu, Zhilan Li, Feng Jiang, Lingzi Ouyang, Shangjun Wu, Chencheng Fu, Ying Liu, Yuanhong Liu, Yuanliang Yan

**Affiliations:** ^1^ Department of Pharmacy, Xiangya Hospital, Central South University, Changsha, China; ^2^ National Clinical Research Center for Geriatric Disorders, Institute for Rational and Safe Medication Practices, Xiangya Hospital, Central South University, Changsha, China; ^3^ Department of Pathology, Xiangya Hospital, Central South University, Changsha, China; ^4^ Department of Pathology, Xiangya Changde Hospital, Changde, China

**Keywords:** resistance, AKAP12, pan-cancer analysis, immune infiltration, prognosis

## Abstract

The primary or acquired resistance to anti-VEGF inhibitors remains a common problem in cancer treatment. Therefore, identifying potential biomarkers enables a better understanding of the precise mechanism. Through the GEO database, three profiles associated with bevacizumab (BV) resistance to ovarian cancer, glioma, and non-small-cell lung carcinoma, respectively, were collected for the screening process, and two genes were found. A-kinase anchor protein 12 (AKAP12), one of these two genes, correlates with tumorigenesis of some cancers. However, the role of AKAP12 in pan-cancer remains poorly defined. The present study first systematically analyzed the association of AKAP12 with anti-VEGF inhibitors’ sensitivity, clinical prognosis, DNA methylation, protein phosphorylation, and immune cell infiltration across various cancers via bioinformatic tools. We found that AKAP12 was upregulated in anti-VEGF therapy-resistant cancers, including ovarian cancer (OV), glioblastoma (GBM), lung cancer, and colorectal cancer (CRC). A high AKAP12 expression revealed dismal prognoses in OV, GBM, and CRC patients receiving anti-VEGF inhibitors. Moreover, AKAP12 expression was negatively correlated with cancer sensitivity towards anti-VEGF therapy. Clinical prognosis analysis showed that AKAP12 expression predicted worse prognoses of various cancer types encompassing colon adenocarcinoma (COAD), OV, GBM, and lung squamous cell carcinoma (LUSC). Gene mutation status may be a critical cause for the involvement of AKAP12 in resistance. Furthermore, lower expression of AKAP12 was detected in nearly all cancer types, and hypermethylation may explain its decreased expression. A decreased phosphorylation of T1760 was observed in breast cancer, clear-cell renal cell carcinoma, and lung adenocarcinoma. For the immunologic significance, AKAP12 was positively related to the abundance of pro-tumor cancer-associated fibroblasts (CAFs) in various types of cancer. The results of Gene Ontology (GO) and Kyoto Encyclopedia of Genes and Genomes (KEGG) pathway analysis suggested that “cell junction organization” and “MAPK pathway” participated in the effect of AKAP12. Importantly, we discovered that AKAP12 expression was greatly associated with metastasis of lung adenocarcinoma as well as differential and angiogenesis of retinoblastoma through investigating the single-cell sequencing data. Our study showed that the dual role of AKAP12 in various cancers and AKAP12 could serve as a biomarker of anti-VEGF resistance in OV, GBM, LUSC, and COAD.

## Introduction

Globally, cancer incidence and mortality remain rapidly rising. There were an estimated 19.3 million new cancer cases and almost 10.0 million cancer-related deaths worldwide in 2020 (Sung et al., 2021). Excessive abnormal angiogenesis is one of the major characteristics of solid tumors. Vascular endothelial growth factor (VEGF) is a well-recognized angiogenic stimulator, overproduction of which contributes to different types of malignancies. Therefore, VEGF-mediated signaling has rapidly evolved into an important target for anti-angiogenic therapy in oncology (Jászai and Schmidt, 2019). For instance, bevacizumab (BV) is a recombinant humanized monoclonal antibody that selectively neutralizes VEGF and exerts an anti-angiogenesis effect. Also, it has been approved for the treatment of various malignancies, including colorectal cancer (CRC), cervical cancers, glioblastoma (GBM), non-small-cell lung cancer (NSCLC), ovarian cancer (OV), and renal cell carcinoma (Mascarenhas et al., 2019). Despite encouraging efficacy demonstrated by anti-angiogenic agents targeting VEGF, resistance during prolonged drug treatments emerges as a limiting feature (Filippelli et al., 2021). Consequently, exploring biomarkers that can predict resistance to anti-VEGF inhibitors is of relevance to improving cancer patients’ prognosis.

A-kinase (PRKA) anchor protein 12 (AKAP12), also known as AKAP250, Gravin, or SSeCKS, was initially identified as a cytoplasmic antigen in sera derived from myasthenia gravis patients (Gordon et al., 1992). AKAP12, belonging to the kinase scaffolding protein family, performs its function by anchoring protein kinase A and protein kinase C to the plasma membrane (Radeva et al., 2014). Recently, the divergent functions of AKAP12 in cancers have been studied extensively. On the one hand, AKAP12 is recognized as a tumor suppressor, whose diminished expression in cancer cells is accompanied by an enhanced invasive and metastatic phenotype (Gelman and Gao, 2006). The expression of AKAP12 has been verified to be decreased in multiple cancers, including hepatocellular carcinoma (HCC) (Lee et al., 2018), CRC (He et al., 2018), and breast cancer (Soh et al., 2018). On the other hand, augmented expression of AKAP12 in both cisplatin- (Lopez-Ayllon et al., 2014) and paclitaxel-resistant (Bateman et al., 2015) cancer cells indicates a cancer-protective role of AKAP12. However, the roles of AKAP12 in various tumor types are to be elucidated.

In the present study, based on bioinformatics technology, increased expression of AKAP12 was proved to influence the prognosis of BV-treated cancer patients. Thereupon, we performed a pan-cancer analysis to explore the detailed roles of AKAP12 across various cancers. We systematically describe the drug sensitivity, prognostic significance, and the genetic alteration states of AKAP12 among different cancer types. The expression difference, protein phosphorylation, and DNA methylation between tumor and normal tissues were also considered. Moreover, we visualized the relationship between AKAP12 expression and immune infiltration via the TIMER2 database. Also, AKAP12-related biochemical pathways were analyzed to investigate the molecular mechanisms of AKAP12. Finally, single-cell sequencing data were employed to explore relevant cancer cell states of AKAP12. This comprehensive analysis suggests potential molecule mechanisms of AKAP12 in the pathogenesis and prognosis of multiple cancer types, providing some reference for target therapies.

## Materials and methods

### Identification of differential expression genes associated with bevacizumab resistance

We searched GEO (Gene Expression Omnibus, http://www.pubmed.com/geo) to screen the datasets suitable for the study (Clough and Barrett, 2016). The keywords “bevacizumab resistance” and “cancer” were used and only datasets with “expression profile by array” as study type were included. Three datasets associated with BV resistance in cancer were incorporated, including GSE45161 for glioma, GSE180687 for OV, and GSE26644 for NSCLC. GSE45161 contained 2 BV-sensitive and 1 BV-resistant tumor tissue. The former was extracted from NSC11 cell line-derived xenograft models from untreated mice while the latter was from NSC11R cell line-derived xenograft models from untreated mice. GSE180687 contained 4 pairs of BV-sensitive and BV-resistant tumor endothelial cell lines. GSE26644 included 3 pairs of BV-sensitive and BV-resistant tissues. BV-sensitive tissues were extracted from H1975 NSCLC cell-derived xenografts from mice treated with control vehicles and BV-resistant tissues were extracted from H1975 NSCLC cell-derived xenografts from mice treated with BV until progression. Then, gene expression profiles of these datasets were downloaded and analyzed to screen differentially expressed genes (DEGs) between BV-resistant and sensitive samples. *p*-value < 0.05 and fold change (FC) > 1.5 were set as the cutoff value. Subsequently, Venn analysis provided by Omicstudio (https://www.omicstudio.cn/index) was employed to identify the overlapping DEGs among the three datasets. Moreover, GEO datasets associated with anti-VEGF resistance, bearing a minimum sample size of two in each group, were sifted to validate the association of co-DEGs and anti-VEGF resistance. These datasets included GSE64472, GSE86525, and GSE89162. GSE64472 based on the GPL6887 platform contained two vandetanib-resistant and three vandetanib-sensitive tissues, as well as three pairs of cediranib-resistant and cediranib-sensitive tissues, extracted from NSCLC xenografts. GSE86525 contained three pairs of BV-resistant and BV-untreated tumors from colon carcinoma xenografts. GSE19862 contained seven pairs of tissues from BV non-responding and responding patients with CRC. For data processing flow, gene probe expression matrix and matched probe annotation file were extracted from the GEO portal, and probes of AKAP12 and INSIG1 were located according to the probe annotation file. Subsequently, inputting corresponding probes, gene profiles of AKAP12 and INSIG1 were obtained employing the ‘Profile Graph’ module in GEO2R, and boxplots were plotted to apply Graphpad software.

### Prognosis analysis

We first utilized the Kaplan–Meier plotter database (https://kmplot.com/analysis/) to explore the relationship between the expression of co-DEGs and the prognosis of OV patients following treatment with BV (Gyorffy et al., 2012). The prognostic index covers the overall (OS), progression-free (PFS), and post-progression survival (PPS). Then, the prognostic significance of the co-DEGs was validated using two datasets including GSE72951 and GSE72969. Specifically, gene expression data and corresponding clinical information of GSE72951 and GSE72969 were downloaded from GEO, respectively. GSE72951 includes 43 glioblastoma multiforme (GBM) patients receiving BV together with semustine while GSE72969 includes 29 CRC patients jointly treated with BV and other chemotherapy. Both datasets contain complete survival information such as survival time and survival status. We separated the patients into high/low expression groups according to the optimal cut-off value of gene expression. Data processing were performed manually while graphing was performed using the Xiantao tool (https://www.xiantao.love). We also employed GEPIA2 (Gene Expression Profiling Interactive Analysis, version 2, http://gepia2.cancer-pku.cn/#analysis) to acquire the OS and Disease-Free Survival (DFS) significance map data as well as survival plots of AKAP12 among all TCGA tumors (Tang et al., 2019). Cancer patients were dichotomized into high- or low-expression cohorts with the medium expression of AKAP12 as a cutoff value.

### Drug sensitivity analysis

The half-maximal inhibitory concentration (IC50) information of anti-VEGF inhibitors, encompassing sorafenib (Hasskarl, 2010) and cediranib (Sahade et al., 2012) and sunitinib (Sunitinib, 2007), and expression profiles of co-DEGs in cancer cell lines were downloaded from DepMap (the Cancer Dependency Map, https://depmap.org/portal/interactive/) portal (Tsherniak et al., 2017). To visualize the correlation of co-DEGs with IC50 values of anti-VEGF therapy, scatter plots were plotted. We also explored the connection between AKAP12 expression and drug sensitivity by employing the BEST (Biomarker Exploration for Solid Tumors, https://rookieutopia.com/app_direct/BEST/) application. The BEST allowed the evaluation of the clinical drug response with only the expression data of clinical tumor samples. VEGF inhibitors, such as sorafenib, brivanib (Dempke and Zippel, 2010), linifanib (Horinouchi, 2016) were chosen. Subsequently, the association between AKAP12 expression and different VEGF inhibitors was shown as scatter diagrams.

### Genetic alteration analysis

CBioportal (https://www.cbioportal.org) offers a web resource for exploring, visualizing, analyzing, and downloading large-scale cancer genomic data (Cerami et al., 2012). Thereupon, CBioportal was employed to attain the data regarding alteration frequency, mutation type, copy-number alteration (CNA), mutated site information as well as the three-dimensional (3D) structure of the protein structure among all TCGA cancer types. Furthermore, survival data encompassing DFS, Disease-Specific Survival (DSS), OS, and PFS were compared for all TCGA tumors, with or without AKAP12 genetic alteration.

### Gene expression analysis

We applied the Timer2 (Tumor Immune Estimation Resource, version 2, http://timer.cistrome.org/) to explore the expression profiling of AKAP12 between tumor and adjacent normal tissues (Li et al., 2020). For those tumors without or merely with limited numbers of normal tissues such as TCGA-adenoid cystic carcinoma (ACC) and so on, GEPIA2 was used for visualizing the AKAP12 expression difference between cancerous and corresponding normal tissues of GTEx (Genotype-Tissue Expression) database. The criteria were set as follows: *p*-value cut-off = 0.05, log2FC cutoff = 1 and “Match TCGA normal and GTEx data”. UALCAN (http://ualcan.path.uab.edu), which is an interactive web resource, can easily conduct in-depth analyses of TCGA gene expression (Chandrashekar et al., 2017). We compared the expression difference of the total or phosphoprotein of AKAP12 in primary cancer and normal tissues, separately. The protein expression analysis was performed using data from the CPTAC (Clinical proteomic tumor analysis consortium) dataset. Moreover, the DNA methylation of AKAP12 was also explored through UALCAN database. AKAP12 expression difference at the transcriptional level was further validated using the TNMplot database (https://tnmplot.com/analysis/) (Á and Győrffy, 2021). Additionally, to further estimate differences in AKAP12 expression at the protein level, we attained immunohistochemistry (IHC) images of AKAP12 proteomic expression in normal and four tumor tissues, including breast invasive carcinoma (BRCA), colon adenocarcinoma (COAD), uterine corpus endometrial carcinoma (UCEC), and ovarian Serous Cystadenocarcinoma from the HPA (Human Protein Atlas, http://www.proteinatlas.org/) (Uhlén et al., 2015). Consistent results from the two databases jointly substantiate the AKAP12 expression difference. Furthermore, we used the TISIDB (Tumor and Immune System Interaction Database, http://cis.hku.hk/TISIDB/index.php) to investigate the relationship between AKAP12 and pathological stages, as well as histological grades of tumors (Ru et al., 2019).

### Immune infiltration analysis

TIMER2 resource was utilized to visualize the relationship between AKAP12 expression and immune infiltrates in tumors of TCGA. B cell, cancer-associated fibroblast (CAF), CD8^+^ T cell, Treg cell, natural killer (NK) cell, macrophage, monocyte, myeloid dendritic cell, and neutrophil were selected for in-depth analysis. The EPIC, MCPCOUNTER, TIDE, XCELL, TIMER, CIBERSORT, CIBERSORT-ABS as well as QUANTISEQ algorithms were applied for estimations.

### A-kinase anchor protein 12-related gene enrichment analysis

The protein–protein interaction (PPI) networks of AKAP12 were acquired via the STRING database (https://string-db.org/) (von Mering et al., 2003). The basic parameters were as follows: minimum required interaction score [low confidence (0.150)], the meaning of network edges (evidence), maximum number of interactors to show (no more than 50 interactors), and active interaction sources (experiments). We then obtained the top 100 AKAP12-related genes through GEPIA2 based on the datasets of all tumor and normal tissues of the TCGA project. Subsequently, a pairwise gene-gene Pearson correlation analysis was conducted to explore the association between AKAP12 and the topsix related genes. The *p*-value and correlation coefficient (R) were exhibited in the plot. What’s more, TIMER2 was employed to create the heatmap data of the top six AKAP12-associated genes, which indicated both the *p*-value and partial correlation (cor) in Spearman’s rank correlation test after purity adjustment. Two sets of data were integrated and filtered to conduct Gene ontology (GO) and Kyoto encyclopedia of genes and genomes (KEGG) pathway analysis. Specifically, we uploaded the gene lists to the Xiantao tool to acquire the functional annotation chart data. Enrichment analysis was conducted with R packages “ClusterProfiler” while enrichment pathways were visualized via the “ggplot2” R packages.

### Single-cell sequencing data analysis

CanserSEA (http://biocc.hrbmu.edu.cn/CancerSEA/home. jsp) is a dedicated database developed for comprehensively exploring cancer cell function at the single-cell level (Yuan et al., 2019). We investigated the association between AKAP12 expression and distinct cancer functional states according to single-cell sequencing data extracted from CancerSEA and plot a heatmap. Only significantly different results with a correlation coefficient greater than 5 were shown. Also, the t-SNE maps of all individual cells were acquired simultaneously from the CancerSEA website.

### Statistical analysis

To compare the survival curve, the log-rank tests were performed to calculate the hazard ratio (HR) and log-rank *p*-value. Differential expression of AKAP12 among tumor and normal tissues was explored by applying the default Wilcoxon’s test or one-way analysis of variance (ANOVA). Pearson’s correlation was used to evaluate the relationship between the two groups. P < 0.05 was considered statistically significant.

## Results

### The differentially expressed genes between bevacizumab-resistant and sensitive groups

**FIGURE 1:**
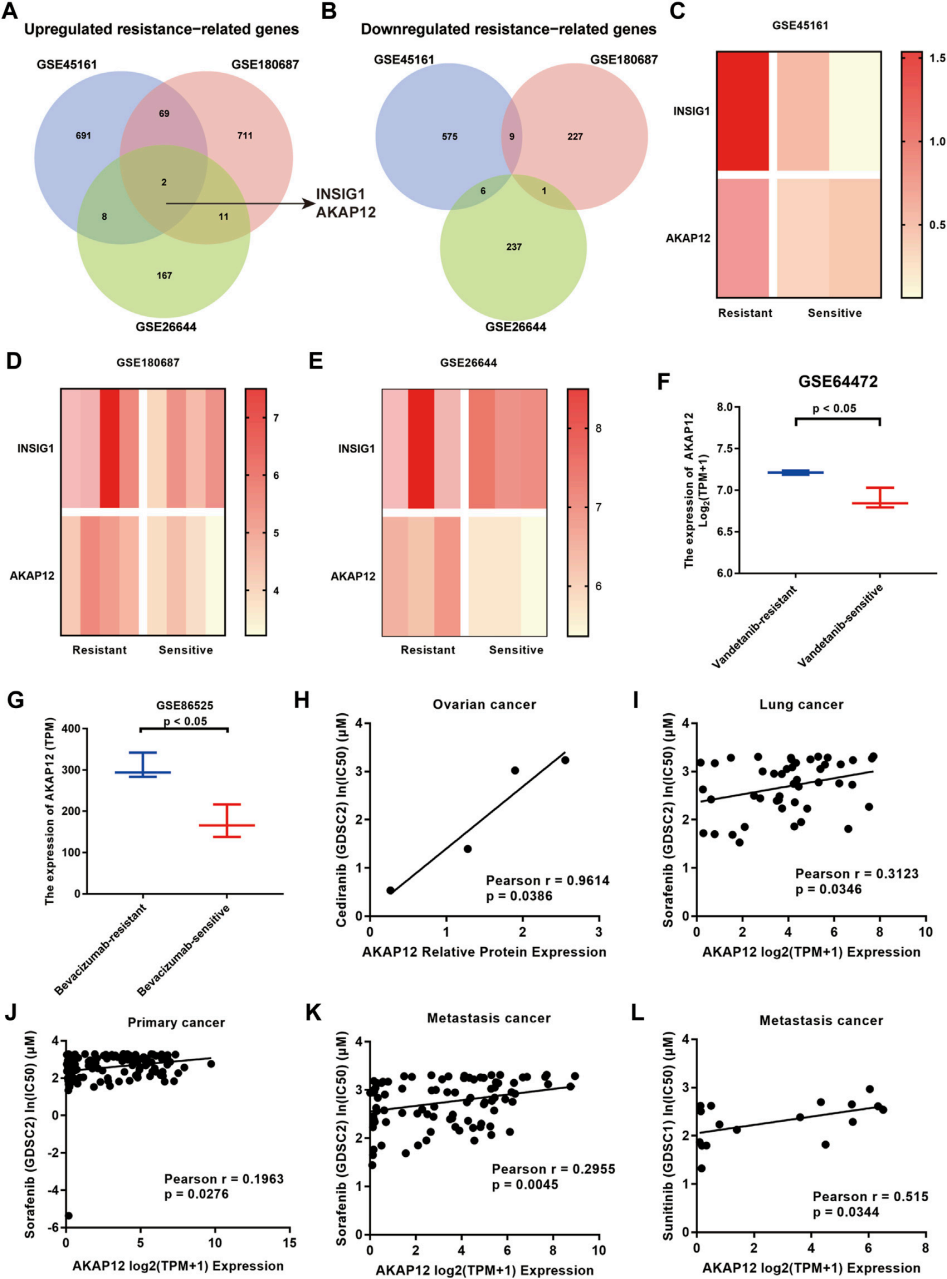
Identification of 2 overlapping differentially expressed genes (DEGs) from 3 GEO datasets (GSE45161 of glioma, GSE180687 of OV, and GSE26644 of NSCLC). **(A)** Two common DEGs, namely, INSIG1 and AKAP12, were identified to be overexpressed in three datasets. **(B)** None of the overlapping DEGs was identified to be underexpressed in the three datasets. **(C–E)** Heatmaps of two common DEGs in the resistant group and sensitive group. **(F)** AKAP12 expression was significantly elevated in vandetanib-resistant NSCLC compared to vandetanib-sensitive NSCLC tissues based on GSE64472. **(G)** Relative to bevacizumab-sensitive tissues, AKAP12 expression was increased in bevacizumab-resistant colon cancer tissues in GSE86525. Scatter plots of the association between AKAP12 expression and IC50 values of cediranib in ovarian cancer **(H)**, IC50 values of sorafenib in lung cancer **(I)**, primary pan-cancer **(J)**, and metastasis pan-cancer **(K)**, as well as IC50 values of sunitinib in metastasis pan-cancer **(L)**.

We screened DEGs between BV-resistant and sensitive groups via analyzing the gene expression profiles of 3 GEO datasets, based on the cutoff criteria of *p* < 0.05 and FC > 1.5. We identified 770 upregulated genes and 590 down-regulated genes in BV-resistant glioma tissues in GSE45161, 793 upregulated genes, 238 down-regulated genes in BV-resistant OV cell lines in GSE180687, 188 upregulated genes, and 244 down-regulated genes in BV-resistant NSCLC tissues in GSE26644, respectively (Supplementary Table S1). After analysis, employing the GEO2R tool and Venn diagrams, two upregulated BV-resistance-related genes INSIG1 and AKAP12 were identified. The Venn diagram is displayed in Figure 1A and Figure 1B. The heatmap showed the enhanced expression of the two overlapping DEGs in BV-resistant cancer (Figures 1C–E). Using two GEO datasets (GSE64472 and GSE86525), significantly increased the expression of AKAP12 was validated in vandetanib-resistant NSCLC (Figure 1F) and BV-resistant colon cancer (Figure 1G). Similarly, according to GSE64472 and GSE19862, a tendency towards elevated levels of AKAP12 was also observed in cediranib-resistant NSCLC (Supplementary Figure S1A) and BV non-responding CRC (Supplementary Figure S1B). These analyses, yet, failed to reach statistical significance, perhaps owing to small sample sizes. As for INSIG1, prominently upregulated INSIG1 expression was validated in vandetanib-resistant NSCLC (Supplementary Figure S1C). An upregulation trend of INSIG1 was also seen in cediranib-resistant NSCLC (Supplementary Figure S1D), BV-resistant colon cancer (Supplementary Figure S1E), and BV non-responding CRC (Supplementary Figure S1F). Additionally, the association of AKAP12 expression and anti-VEGF sensitivity was analyzed by depmap portal. Higher IC50 values of cediranib, sorafenib, and sunitinib indicated resistance to anti-VEGF therapy. AKAP12 expression was positively correlated with IC50 values of cediranib in OV (Figure 1H). Notably, a significant positive relationship was observed between AKAP12 and IC50 values of sorafenib in lung cancer (Figure 1I), primary pan-cancer (Figure 1J), and metastasis pan-cancer (Figure 1K). Also, AKAP12 expression was correlated with higher IC50 values of sunitinib in metastasis pan-cancer (Figure 1L). Nevertheless, no obvious significance was found between INSIG1 expression and IC50 values of anti-VEGF therapies (Supplementary Figures S1G–J). These findings indicate that cancer tissues with higher AKAP12 expression might correspond to a higher potential for anti-VEGF therapy resistance.

### A-kinase anchor protein 12 exhibits poor prognosis in cancer patients receiving bevacizumab

**FIGURE 2:**
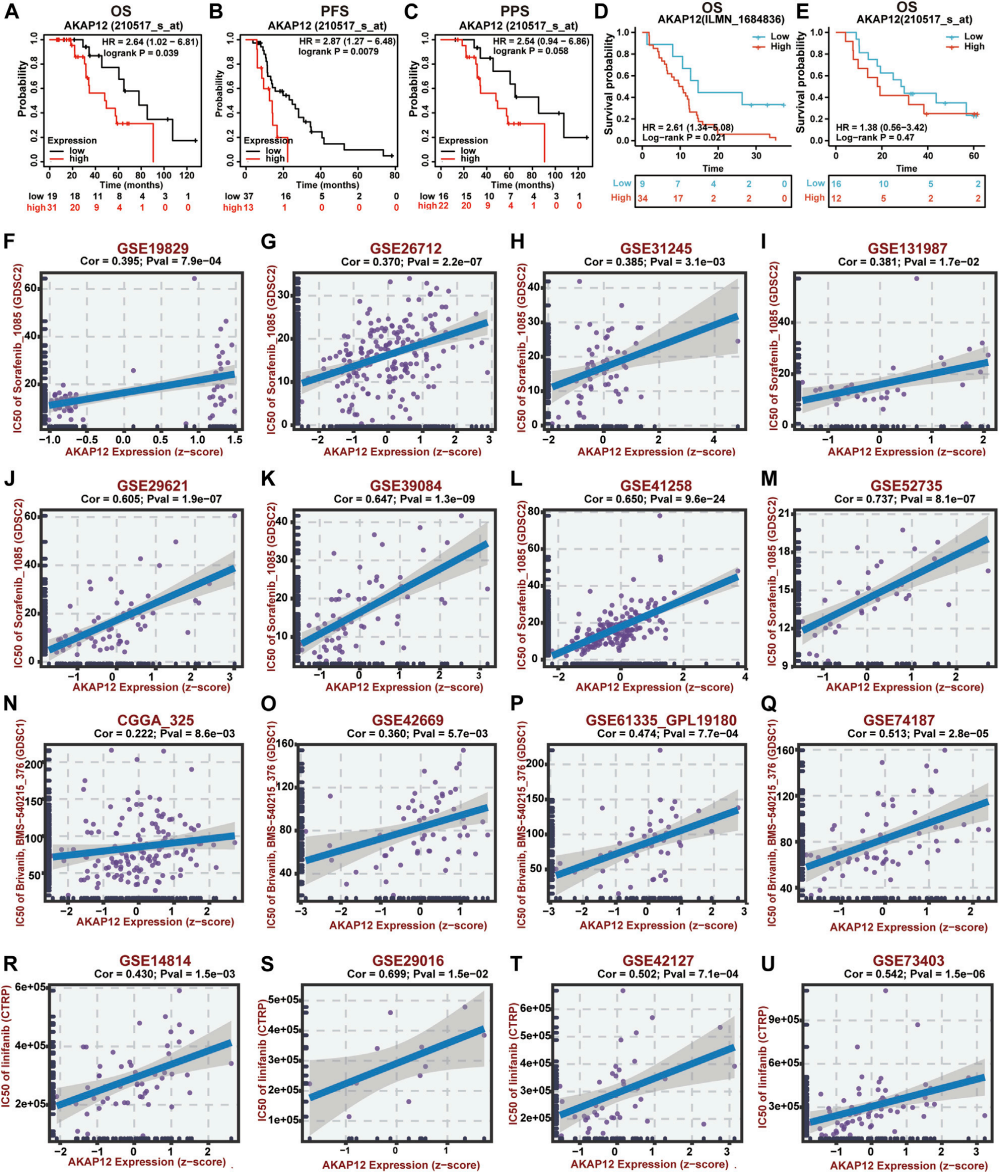
Prognostic value and drug sensitivity of AKAP12 in several cancers. The prognostic value of AKAP12 in OV patients **(A–C)**, GBM patients **(D),** and CRC patients **(E)** following BV treatment. OS, overall survival; PFS, progression-free survival; PPS, post-progression survival. Scatter plot of the association between AKAP12 expression and anti-VEGF therapy sensitivity of OV patients **(F–I)**, CRC patients **(J–M)**, GBM patients **(N–Q)**, and LUSC patients **(R–U)**.

The correlation between the expression of INSIG1 as well as AKAP12 and their corresponding prognosis in cancer patients receiving BV was analyzed applying the Kaplan–Meier plotter database and two GEO datasets. Of note, high AKAP12 expression was significantly associated with poor OS (*p* = 0.039) and PFS (*p* = 0.0079). Meanwhile, BV-treated OV patients with high levels of AKAP12 displayed marginally unfavorable PPS (*p* = 0.058) (Figures 2A–C). Consistently, enhanced expression of AKAP12 was related to dismal OS (*p* = 0.021) of GBM patients who received BV and semustine (Figure 2D). CRC patients were also found to have worse OS (*p* = 0.47) following treatment with BV and other chemotherapy, although statistical significance was not achieved because of the limited number of samples (Figure 2E). In terms of INSIG1, BV-treated OV patients who have increased expression of INSIG1 exhibit a remarkable association with poor OS (*p* = 0.02) and PPS (*p* = 0.01), reversely, a slight relationship with ideal PFS (*p* = 0.052) (Supplementary Figure S2A). However, there was no obvious correlation between the expression of INSIG1 and clinical outcomes of both GBM (*p* = 0.25) (Supplementary Figure S2B) and CRC patients (*p* = 0.16) (Supplementary Figure S2C) who are administrated with BV. These results thereby clearly implied that AKAP12 expression was significantly linked with dismal prognosis in certain types of cancer patients receiving BV.

### The correlation between A-kinase anchor protein 12 expression and drug sensitivity

Using the BEST database, we further investigated the possible correlation between AKAP12 expression and drug sensitivity of VEGF receptor inhibitors in OV, GBM, CRC, and lung squamous cell carcinoma (LUSC) patients. Notably, AKAP12 expression showed a positive correlation with the IC50 values of sorafenib in OV (Figures 2F–I) and CRC (Figures 2J–M). Also, significantly positive correlations were found between AKAP12 expression and IC50 values of bravanib in GBM (Figures 2N–Q), as well as IC50 values of linifanib in LUSC (Figures 2R–U). These results indicated that patients with high AKAP12 levels may be resistant to the treatment of anti-VEGF inhibitors.

### The relationship between gene expression of A-kinase anchor protein 12 and survival in pan-cancer

**FIGURE 3:**
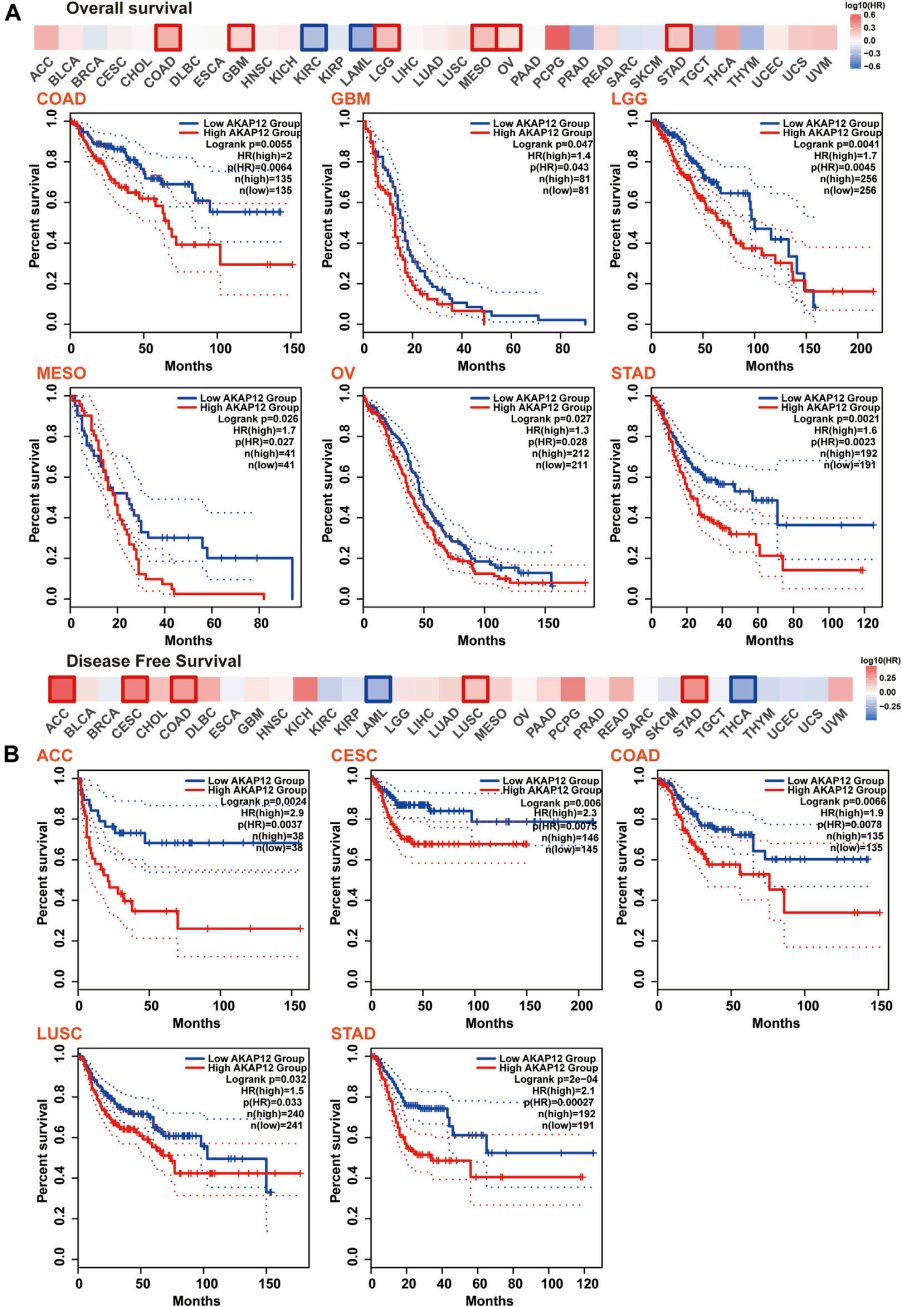
Correlation between AKAP12 expression and patient survival across all TCGA tumors. Correlation between AKAP12 expression and overall survival **(A)**, disease-free survival **(B)** were evaluated among TCGA cancers employing GEPIA2. Only significantly negative correlations between AKAP12 expression and prognosis were visualized by Kaplan-Meier curves.

Next, we tended to focus on understanding how AKAP12 expression related to the prognosis of patients afflicted with cancer. We stratified cancer patients into high-and low-AKAP12 expression groups according to medium expression value, and subsequently, TCGA and GEO datasets were utilized to explore the association between AKAP12 expression and the prognosis of distinct tumor patients. The results indicated that high expression of AKAP12 was correlated with worse OS for colon adenocarcinoma (COAD) (*p* = 0.0055), glioblastoma multiforme (GBM) (*p* = 0.047), brain low-grade glioma (LGG) (*p* = 0.0041), mesothelioma (MESO) (*p* = 0.026), ovarian serous cystadenocarcinoma (OV) (*p* = 0.027), stomach adenocarcinoma (STAD) (*p* = 0.0021) (Figure 3A). DFS analysis data showed that the reinforced expression of AKAP12 was related to the detrimental prognosis of adrenocortical carcinoma (ACC) (*p* = 0.0024), cervical squamous cell carcinoma and endocervical adenocarcinoma (CESC) (*p* = 0.006), COAD (*p* = 0.0066), LUSC (*p* = 0.032), STAD (*p* = 2e-04) (Figure 3B). Kaplan–Meier survival analyses revealed an association between high expression levels of AKAP12 and adverse OS (*p* = 8.7e-08 and *p* = 1.2e-09, respectively), and PPS (*p* = 0.015 and *p* = 4.3e-08, respectively) for OV and gastric cancer (GC), poor PFS for OV (*p* = 2.4e-06), and poor first progression (FP) for GC (*p* = 4.6e-08) (Supplementary Figure S3).

### Genetic alteration analysis

**FIGURE 4:**
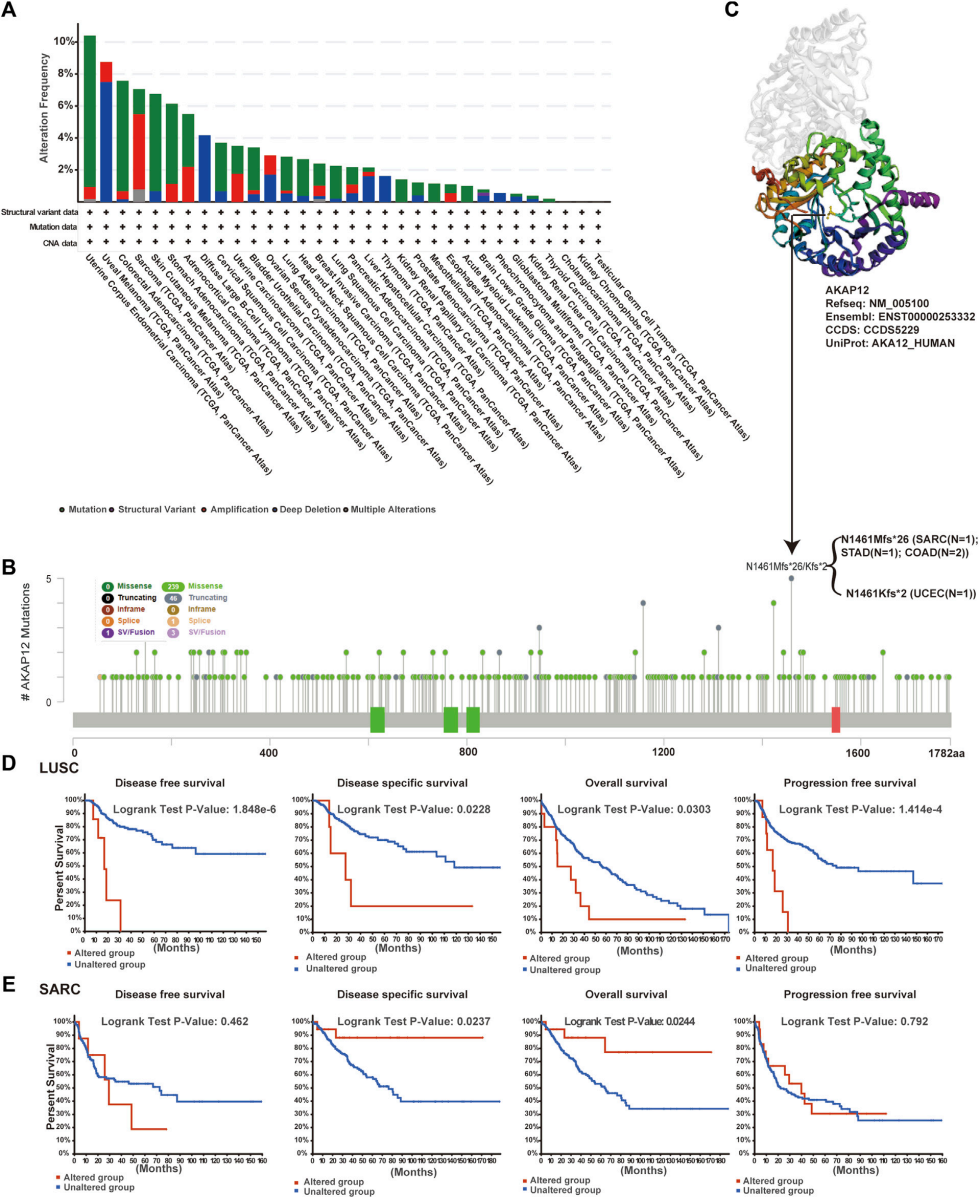
Mutation status of AKAP12 in TCGA tumors. Mutation states of AKAP12 in TCGA tumors were analyzed utilizing the cBioPortal portal. Genetic alteration type and frequency **(A)**, and mutation site **(B)** are exhibited. **(C)** The mutation site (N1461) was displayed in the 3D structure of AKAP12. **(D–E)** Characterization of the relationship between AKAP12 alteration status and disease-free, disease-specific, overall, and progression-free survival of LUSC and SARC.

The genetic alteration profiling of AKAP12 in different tumor samples within the TCGA cohorts was explored. According to our analysis, the highest alteration frequency of AKAP12 appeared for Uterine Corpus Endometrial Carcinoma (UCEC) cases with “mutation” as the predominant type. Uveal Melanoma (UVM) had the highest incidence of the “deep deletion” type of CNV, yielding a frequency of ∼8% (Figure 4A). Then, the mutation type, sites as well as case number of the AKAP12 genetic alteration are shown in Figure 4B. We found that missense mutation of AKAP12 was the primary type of genetic alteration and the N1461Mfs*26/Kfs*2 alteration, a truncation mutation, was detected in one case of sarcoma (SARC), one case of STAD, one case of UCEC, and two cases of COAD. The N1461 site in the 3D structure of AKAP12 protein was shown in Figure 4C. To examine whether there is an association between certain genetic alterations of AKAP12 and clinical outcomes of patients, we systematically analyzed and correlated these with diverse cancer types. The data in Figure 4D indicated that LUSC patients with altered AKAP12 have worse prognosis in DFS (*p* = 1.848e-6), DSS (*p* = 0.0228), OS (*p* = 0.0303), and PFS (*p* = 1.414e-4) compared with cases without AKAP12 alterations. Conversely, the data of Figure 4E suggest that SARC patients with altered AKAP12 displayed better prognosis in DSS (*p* = 0.0237), OS (*p* = 0.0244), but not DFS (*p* = 0.462), PFS (*p* = 0.792), relative to cases without AKAP12 alterations.

### Gene expression analysis

First, we employed the TIMER2 to assess the expression pattern of AKAP12 among varieties of TCGA tumors. As shown in Supplementary Figure S4A, the expression levels of AKAP12 in tumor tissues of bladder urothelial carcinoma (BLCA), BRCA, CESC, COAD, head, and neck squamous cell carcinoma (HNSC), kidney chromophobe (KICH), liver hepatocellular carcinoma (LIHC), lung adenocarcinoma (LUAD), LUSC, prostate adenocarcinoma (PRAD), rectum adenocarcinoma (READ), thyroid carcinoma (THCA), UCEC (*p* < 0.05) were lower as relative to the corresponding control tissues. Of note, a few tumor types including cholangiocarcinoma (CHOL), esophageal carcinoma (ESCA), GBM, kidney renal clear cell carcinoma (KIRC), kidney renal papillary cell carcinoma (KIRP), pancreatic adenocarcinoma (PAPD), pheochromocytoma and paraganglioma (PCPG), and STAD showed no differential expression (*p* > 0.05).

As for the tumors lacking normal tissues in the TCGA repository, we further evaluated the expression differences of AKAP12 between tumor and normal tissues through the GTEx database. We identified that ACC, OV, and uterine carcinosarcoma (UCS) displayed a lower expression in tumor tissues while skin cutaneous melanoma (SKCM) and thymoma (THYM) showed a higher expression (*p* < 0.05). For other tumors, covering lymphoid neoplasm diffuse large B-cell lymphoma (DLBC), acute myeloid leukemia (LAML), LGG, MESO, SARC, testicular germ cell tumors (TGCT), and UVM, we did not attain significant differences (Supplementary Figure S4B). In summary, we detected that AKAP12 expression was reduced in most tumor types. Apart from transcription, we also analyzed AKAP12 at a protein level utilizing the large-scale proteomic data extracted from the CPTAC dataset. We noted that AKAP12 total protein level was significantly decreased in breast cancer, colon cancer, HNSC, hepatocellular carcinoma (HCC), LUAD, OV, and UCEC tissues in comparison with normal tissues (*p* < 0.05) (Supplementary Figure S4C).

Moreover, we analyzed IHC results supplied by the HPA portal and compared the results to AKAP12 expression data from TNMplot. Analysis results of these two databases were coincident with one another. Normal breast, colon, endometrium, and ovary tissues showed medium or strong staining, while tumor tissues showed negative or weak staining (Supplementary Figure S4D).

**FIGURE 5:**
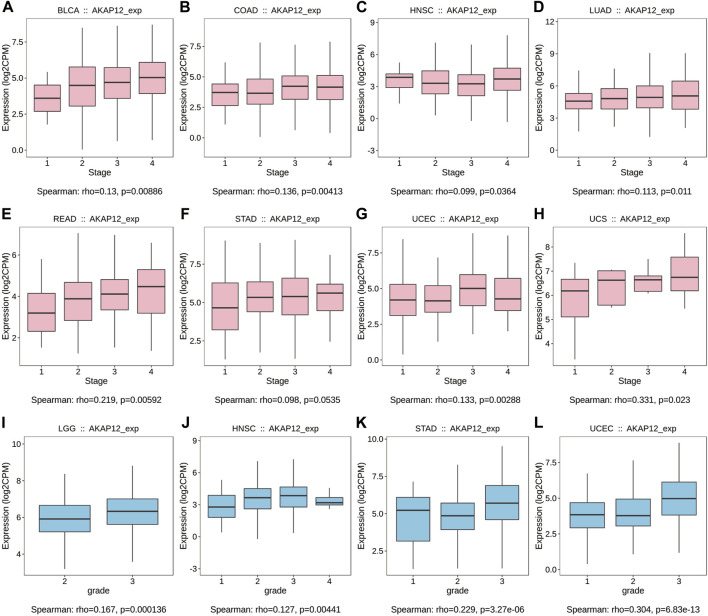
Correlation between AKAP12 expression and stages as well as grades among various TCGA tumors based on the TISIDB database. Correlation of AKAP12 with stages of BLCA **(A)**, COAD **(B)**, HNSC **(C)**, LUAD **(D)**, READ **(E)**, STAD **(F)**, UCEC **(G)**, UCS **(H)**. Correlation of AKAP12 expression with grades of LGG **(I)**, HNSC **(J)**, STAD **(K)**, and UCEC **(L)**.

We further exploited the TISIDB tool to investigate the relationship between AKAP12 expression and pathological stages and histological grades. It is worth noting that AKAP12 expression was positively associated with a pathological stage in BLCA, COAD, HNSC, LUAD, READ, STAD, UCEC, and UCS (Figures 5A–H). Also, with enhanced AKAP12 expression, higher histological tissue grades were shown in LGG, HNSC, STAD, and UCEC (Figures 5I–L).

DNA methylation is a well-recognized epigenetic mechanism impacting gene expression, and abnormal DNA methylation patterns have been characterized in cancer (Dmitrijeva et al., 2018). We evaluated the relationship between AKAP12 expression and methylation levels of AKAP12 promoter in multiple cancers via UALCAN website. A significant elevation in the methylation level of AKAP12 was observed in BLCA, BRCA, CESC, CHOL, COAD, ESCA, HNSC, KIRP, LIHC, LUAD, LUSC, PAAD, PRAD, READ, THCA, and UCEC tissues relative to normal tissues (*p* < 0.05). Reversely, the methylation levels of AKAP12 in PCPG and TGCT were prominently reduced (*p* < 0.05) (Supplementary Figure S5A).

Among various kinds of post-translational modifications (PTMs), protein phosphorylation is the most broadly investigated one, which contributes to the occurrence and development of cancer through diverse aspects (Mao et al., 2021). Thereupon, we examined alteration in AKAP12 phosphorylation between primary tumor versus normal tissues. We analyzed three types of tumors encompassing BRCA, KIRC, and LUAD in more detail utilizing the CPTAC dataset. AKAP12 phosphorylation sites with significant differences are summarized in Supplementary Figure S5B. We detected that the S651 locus of AKAP12 showcases lower phosphorylation levels for BRCA and LUAD, and higher phosphorylation levels for KIRC, followed by decreased phosphorylation levels of the T1760 locus for BRCA, KIRC, and LUAD (Supplementary Figure S5C).

### Immune infiltration analysis

**FIGURE 6:**
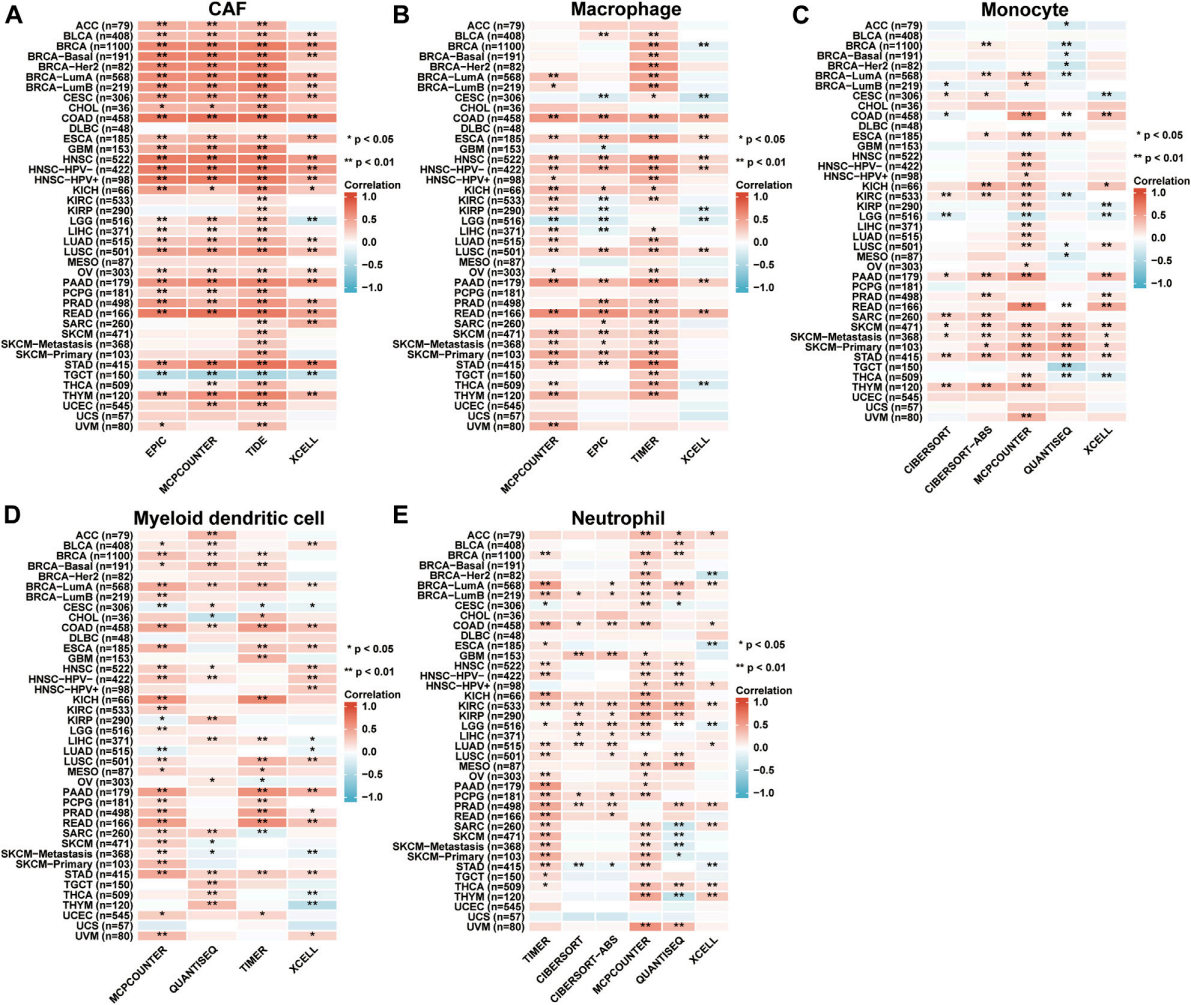
Immune infiltration analysis based on TIMER2 database. The correlation between the AKAP12 expression and infiltration of cancer-associated fibroblasts **(A)**, macrophages **(B)**, monocytes **(C)**, myeloid dendritic cells **(D)**, and neutrophils **(E)**. Among all TCGA tumors according to the EPIC, MCPCOUNTER, TIDE, XCELL, TIMER, CIBERSORT, CIBERSORT-ABS, as well as QUANTISEQ algorithms were analyzed. The red color suggests a positive association (0-1), whereas the blue color indicates an inverse association (-1-0). The association with *p* < 0.05 is considered statistically significant.

According to the EPIC, MCPCOUNTER, TIDE, XCELL, TIMER, CIBERSORT, and CIBERSORT-ABS as well as QUANTISEQ algorithms, the relationship between AKAP12 expression and the infiltration levels of cancer-associated fibroblasts (CAF), macrophages, monocytes, myeloid dendritic cells, neutrophils were estimated across distinct TCGA cohort tumors. Association with the consistent trend in most algorithms was perceived to be credible. Importantly, we discovered that in BLCA, BRCA, BRCA-Basal, BRCA-LumA, BRCA-LumB, CESC, COAD, ESCA, HNSC, HNSC-HPV-, HNSC-HPV+, KICH, LUAD, LUSC, OV, PAAD, PRAD, READ, STAD and THYM, the expression levels of AKAP12 displayed positive association with the estimated infiltration value of CAFs, while in TGCT it showed a negative correlation. We also detected positive associations between AKAP12 expression and the macrophage infiltration for COAD, ESCA, HNSC, HNSC-HPV-, LUSC, PAAD, and READ, the monocyte infiltration for SKCM, SKCM-metastasis, and STAD, myeloid dendritic cell infiltration for BRCA-LumA, COAD, and STAD, as well as the neutrophil infiltration for KIRC (Figure 6). However, there was no apparent connection between the expression levels of AKAP12 and the infiltration of B cells, NK cells, CD8^+^ cells, and Treg cells among all TCGA tumors (Supplementary Figure S6).

### Enrichment analysis of A-kinase anchor protein 12-related partners

**FIGURE 7:**
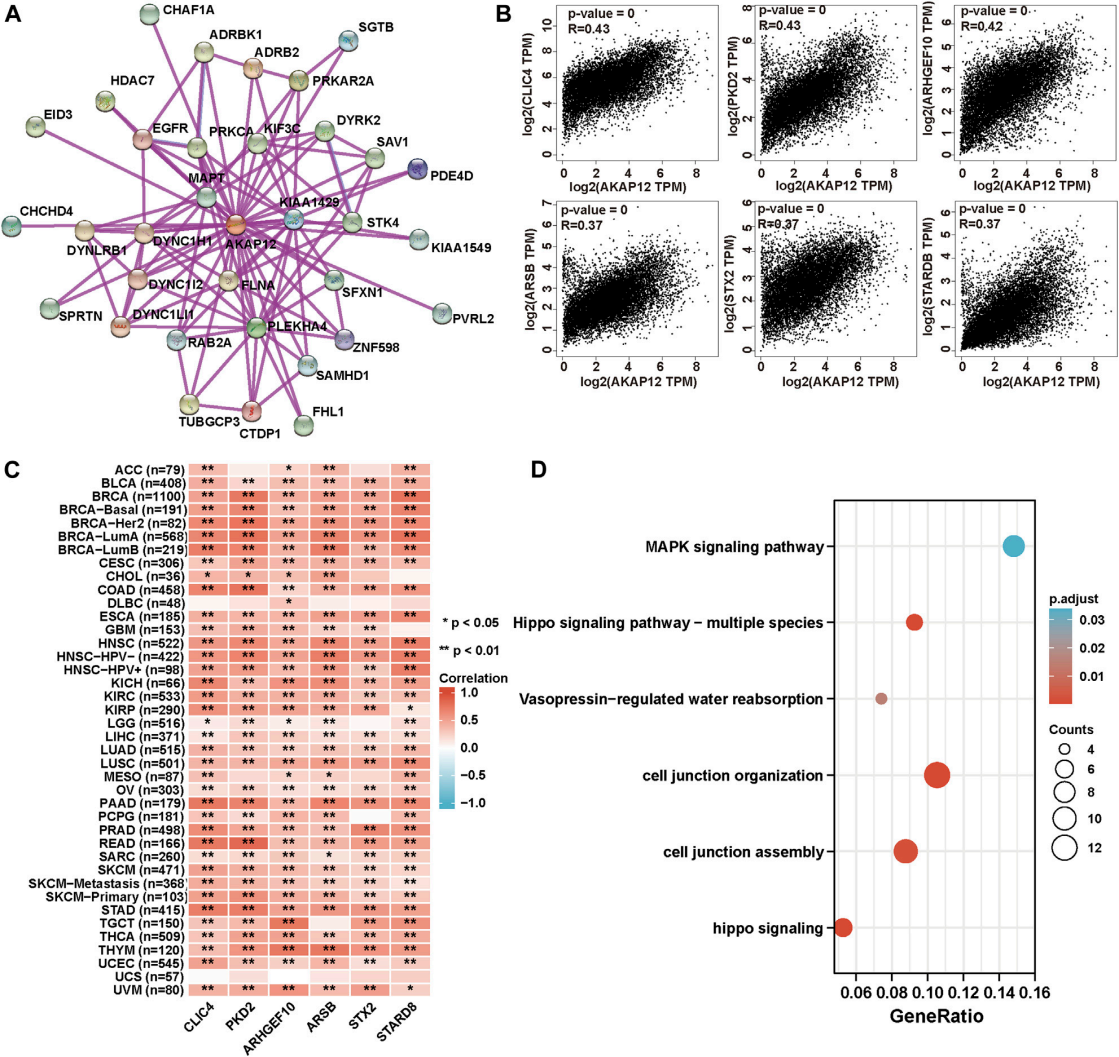
AKAP12-related gene enrichment and pathway analysis. **(A)** Protein network graph of experimentally determined AKAP12-binding proteins generated using STRING. Colored nodes represent the individual proteins identified. **(B)** Relationship between AKAP12 expression and representative genes (CLIC4, PKD2, ARHGEF10, ARSB, STX2, STARD8) of the top AKAP12-related genes within TCGA projects as determined via the GEPIA2 portal. **(C)** Heatmap representation of the expression association between AKAP12 and CLIC4, PKD2, ARHGEF10, ARSB, STX2, and STARD8 in all TCGA tumors. **(D)** GO-KEGG analysis according to the AKAP12-binding and interacted genes.

To acquire a better cognition regarding the molecular mechanisms of AKAP12 underlying tumorigenesis and development, we performed a range of pathway enrichment analysis on AKAP12-interacting proteins and genes bearing a similar expression pattern to AKAP12. A total of 34, experimentally detected AKAP12-binding proteins were identified using the STRING tool. Figure 7A exhibits the interaction network of these 34 proteins. Then, we applied the GEPIA2 to acquire the first 100 genes which showed a similar expression pattern to AKAP12 in different cancer types in TCGA. The expression of AKAP12 was positively related with that of chloride intracellular channel 4 (CLIC4) (R = 0.43), polycystic kidney disease 2 (PKD2) (R = 0.43), Rho guanine nucleotide exchange factor 10 (ARHGEF10) (R = 0.42), arylsulfatase B (ARSB) (R = 0.37), shiga toxin type 2 (STX2) (R = 0.37), and StAR related lipid transfer domain containing 8 (STARD8) (R = 0.37) genes (all *p* < 0.001) (Figure 7B). Heatmap data showcased that AKAP12 had a strong positive relationship with the abovementioned six genes in the majority of cancer types (Figure 7C). We integrated the two datasets for GO and KEGG enrichment analyses. The results revealed that “cell junction organization” and “MAPK signaling pathway” were among the top hits, indicating these pathways in the impact of AKAP12 on tumor pathogenesis (Figure 7D).

### Single-cell sequencing data analysis

**FIGURE 8:**
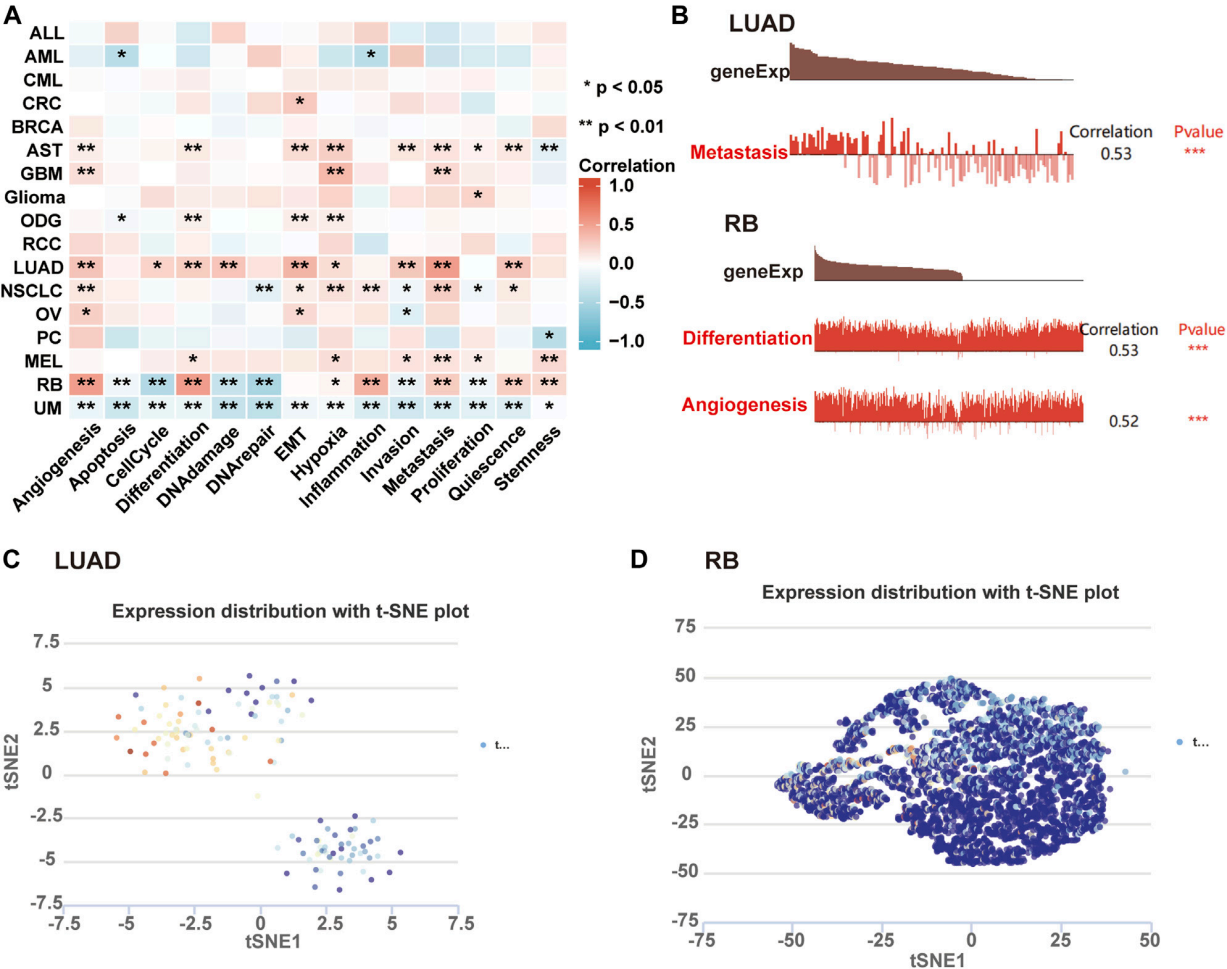
Expression pattern of AKAP12 in single-cell sequencing and its relation to tumor functional status. **(A)** Heatmap representation of the correlation of AKAP12 expression and distinct tumor functional states based on CancerSEA (*p < 0.05; **p < 0.01; ***p < 0.001). **(B)** Association between the expression of AKAP12 and three significantly different functional states (***p < 0.001). T-SNE diagram illustrated AKAP12 expression profiles in single cells of LUAD **(C)** and RB **(D)** samples, separately.

We further investigated the roles of AKAP12 in tumor functional status through the CancerSEA website. The heatmap visualized the significant relationship between AKAP12 expression with distinct tumor states (Figure 8A). In particular, the significantly different results with a correlation coefficient greater than five exhibited a positive association between AKAP12 expression and metastasis in LUAD, as well as angiogenesis and differential in retinoblastoma (RB) (Figure 8B). AKAP12 expression profiles were showcased in single cells of LUAD (Figure 8C), and RB (Figure 8D) by a T-SNE diagram. The results indicated that AKAP12 might play a paramount role in the progress of cancer progression.

## Discussion

Anti-angiogenic therapy is a promising approach to combat cancers that restrains the nutrient and oxygen supply to cancer cells via decreasing the vascular network and avoiding the formation of new blood vessels (Lopes-Coelho et al., 2021). BV (Avastin), a recombinant humanized monoclonal antibody against VEGF, received FDA approval as the first anti-angiogenic agent for the treatment of metastatic CRC in 2004 (Hurwitz et al., 2004). Despite diverse types of VEGF pathway inhibitors which contribute to ameliorating the survival of most cancer patients, some patients gain little or no beneficial effects from them due to the resistance to these anti-VEGF inhibitors (Itatani et al., 2018). In this study, we screen three expression arrays associated with the BV resistance of glioma, OV, and NSCLC from the GEO database and identified two DEGs including INSIG1 and AKAP12, both upregulated in the BV-resistant cancer group. Moreover, the elevated expression of AKAP12 was also validated in vandetanib-resistant NSCLC and BV-resistant colon cancer. Higher AKAP12 expression indicates a higher likelihood of anti-VEGF therapy resistance in cancers, especially in OV and lung cancer. Previous reports illustrated that AKAP12 correlated to VEGF-mediated angiogenesis, while there are no reports on the association of INSIG1 with VEGF. Furthermore, the high expression of AKAP12 was evidenced to have detrimental prognoses in OV, GBM, and CRC patients receiving BV. Also, the drug sensitivity analysis revealed a negative association between AKAP12 expression and the sensitivity of certain cancer types towards anti-VEGF inhibitors. Therefore, our results suggested that AKAP12 might be involved in cancer resistance to anti-VEGF inhibitors, especially for BV.

Regarding the function of AKAP12 in anti-VEGF therapy, one possible molecular mechanism is the downregulation of VEFG signaling. Multiple lines of evidence have demonstrated that AKAP12 functioned as an antagonist of VEGF, associated with the repression of angiogenesis (Gelman, 2010). But contradictory results also demonstrated that suppression of VEGF signaling could also result in an invasive tumor phenotype and subsequent resistance to anti-VEGF therapy (Soda et al., 2013). The overexpression of AKAP12 has been validated to facilitate migration (Grove and Bruchey, 2001). Consistently, Benz et al. (2020) found that AKAP12 was upregulated in actively migrating, VEGF-stimulated endothelial cells, and silencing of AKAP12 contributed to the defects of migration and proliferation, as well as the attenuation of angiogenesis *in vitro* and *in vivo*. Thus, whether AKAP12 could promote anti-VEGF therapy resistance via elevating the migratory capacity of cancer cells need to be elucidated. In our study, AKAP12 expression was positively connected with macrophage infiltration in several cancer types. Intriguingly, a recent study by Dalton et al. (2017) found that the decreased expression of macrophage VEGFR-1 and VEGFR-3 accompanied increased activation of alternative angiogenic pathways, promoting escape from anti-VEGF therapy, suggesting that AKAP12 mediated anti-VEGF therapy resistance maybe through interplaying with macrophages.

The analysis of protein profiling provides valuable insights into developing prognostic biomarkers for early diagnosis and helps make therapeutic plans for specific cancer types (Borrebaeck, 2017). In our study, AKAP12 was downregulated in almost all cancers but was negatively associated with anti-VEGF therapy sensitivity in OV, GBM, CRC, and LUSC. Moreover, AKAP12 was also positively associated with clinical stages of BLCA, COAD, HNSC, LUAD, READ, STAD, UCEC, and UCS, and positively correlated with grades of LGG, HNSC, STAD, and UCEC. Considering the difference in molecular detail between tumorigenesis and chemosensitivity, our finding suggested that AKAP12 was not an oncogene but could act as a resistance-related gene in tumor progression. Not surprisingly, data from prognosis analysis of AKAP12 predicted worse survival in multiple cancers. In line with our findings, AKAP12 was evidenced to play a pivotal role in ovarian granulosa cells and participated in OV invasion and metastasis (Erlichman et al., 1999; Binder et al., 2013; O'Donnell et al., 2005). The report from Conrads’ group has highlighted elevation in AKAP12 expression accompanied by the attainment of platinum- and taxol-resistance in OV cell lines and that enhanced AKAP12 transcript expression in patients affected by high-grade OV is prominently correlated with poor PFS and OS (Chappell et al., 2012; Bateman et al., 2015). Consistently, AKAP12 involved in tumor metastasis was induced in cisplatin-resistant NSCLC H460 cells (Lopez-Ayllon et al., 2014). Besides, AKAP12 protein levels are higher in CRC cells which hints at a positive role in proliferation (Yildirim et al., 2013), and enhanced expression of AKAP12 is linked with the poor survival of CRC patients (Martinez-Romero et al., 2018). Also, Liu et al. (2009)found that AKAP12 knockdown could reverse dexamethasone-induced rat glioma cell growth arrest via elevating cyclin D1 expression. These findings indicated that AKAP12 acts as a resistance-related function in OV, NSCLC, CRC, and GBM. Considering the negative association with drug sensitivity and predictable capacity for shorter survival, the silencing of AKAP12 in these cancer types might, to some extent, enhance cancer patients’ response to anti-VEGF therapy. What cannot be neglected, however, AKAP12 is highly expressed in normal cells, which suggests that the inhibition of AKAP12 as a therapeutic target may affect normal cells. The way to overcome this toxicity and adverse effects should take more concentration on how to increase the efficiency of targeted cancer therapy. Nowadays, gene-targeting therapies and drug carriers are important research fields in nanomedicine to improve drug delivery. For example, nanoparticles such as RNA delivery vehicles have gained tremendous attention for enhancing therapeutic efficiency and reducing systemic toxicity in cancer treatment (Revia et al., 2019). Nevertheless, we also identified that AKAP12 indicated better prognoses in KIRC, LAML, and THCA, this might be attributed to the difference in genetic heterogeneity and clinical features of different cancer types and subtypes (Meacham and Morrison, 2013). Of note, the AKAP12 expression was found to be diminished in acute leukemia samples, which was associated with an inferior OS (Yildirim et al., 2007; Mostafa et al., 2013). Moreover, the tumor suppressor effects of AKAP12 were also noted in certain cancer types such as BRCA and LIHC. A recent study has demonstrated that AKAP12 expression was reduced in breast cancer and repressed chemotaxis-induced cell migration through regulating F-actin (Soh et al., 2018). Consistently, AKAP12 inhibition could rescue the oncogenic miR-1251-5p knockdown-attenuated HCC cell metastasis (Han et al., 2020). The inconsistent role of AKAP12 suggested the possibility of usage of AKAP12 inhibitors contributed to the strategies of precision medicine in future medical inventions based on variable cancer types and specific drug resistance.

The essential role of somatic mutation was confirmed in drug resistance (Nussinov et al., 2021). In a recent study by Hsu et al. (2018), deleterious mutations in PTPRT/PTPRD were related to BV response states of metastasis CRC patients, with the enrichment of deleterious mutations occurring in non-responders, and patients with deleterious alterations showed a shorter PFS (Hsu et al., 2018). In our study, LUSC patients with AKAP12 alteration exhibited a dismal prognosis, and SARC patients with altered AKAP12 had a better prognosis. Therefore, we hypothesized that there is a certain connection between AKAP12 alteration with drug resistance.

Aberrant DNA methylation patterns, generally oncogenes are activated through promoter hypomethylation whereas tumor suppressor genes are silenced through promoter hypermethylation, have been linked with the varieties of human malignancies (Palanichamy et al., 2016). Moreover, abnormal DNA methylation provides important molecular markers for the early detection, diagnosis, and prognosis of cancer patients (Pan et al., 2018). Several studies have confirmed that decreased expression of AKAP12 in advanced cancer was attributed to upregulated hypermethylation of the 5′ CpG island within the AKAP12 promoter region (Han et al., 2015). Most tumor types in the present study were correlated with the low expression levels of AKAP12 and matched high methylation levels of AKAP12 promoters relative to normal tissues, in the support of both previous studies and the potential role of AKAP12 as a biomarker.

The phosphorylation findings of our study indicated diminished phosphorylation levels of T1760 in BRCA, KIRC, and LUAD. Whether PTM sites show clinical relevance remained to be verified. We also analyzed the pivotal signaling pathways of AKAP12. The results implied that AKAP12 was mainly related to the cell junction pathway and MAPK signaling pathway. Cell-cell junctions play an essential role in maintaining tissue homeostasis during critical cell processes including tissue barrier function, cell proliferation, and migration through connecting cells to each other in tissues (Garcia et al., 2018). The previous study has reported that AKAP12 could induce the formation of the blood-retinal barrier (BRB) via barriergenesis in developing human eyes, and defects in these mechanisms contributed to the disappearance of tight junction proteins, thereby leading to retinal pathologies such as RB (Choi et al., 2007). Notably, MAPK cascades are key signaling elements that can modulate a wide assortment of basic processes composing cell proliferation, differentiation, apoptosis, and stress response (Guo et al., 2020). MAPK signaling cascade is one of the best-characterized pathways in cancer biology, hyperactivation of which contributes to over 40% of human cancer cases (Yuan et al., 2020). Unfortunately, there was currently no report regarding the probable relationship between AKAP12 and MAPK signaling, and further investigations are needed.

The tumor microenvironment (TME) is always linked with diverse responses to immunotherapy as well as different clinical outcomes among cancers (Lei et al., 2020). Infiltration of immune cells within TME generally correlates with the occurrence and development of most cancer types. As an essential stromal cell population of TME, most of CAFs subpopulations typically displayed cancer-promoting effects (Chen et al., 2021). Neutrophils, also representing a considerable part of the tumor stroma, can contribute to cancer initiation, growth, proliferation, or metastatic spread, and high infiltration of neutrophils is associated with detrimental outcomes (Ocana et al., 2017). Notably, a high tumor-associated macrophages (TAMs) infiltration is typically correlated with adverse prognosis, but as greatly plastic cells, macrophages can adopt either anti-tumor or pro-tumor phenotype towards stimuli from TME (Malfitano et al., 2020). Dendritic cells (DCs) represent a small fraction of the TME, yet are an important antitumor component due to their capacity to foster T cell immunity and induce immunotherapy response (Gerhard et al., 2021). Classical monocytes mainly differentiate into pathogenic TAMs, but they can also differentiate into DCs that are necessary for effective adaptive immune responses (Olingy et al., 2019). Our analysis strongly showed that AKAP12 played a vital role in the immune infiltration of various cancer types, especially for tumor-promoting CAFs. Given that AKAP12 was negatively linked with anti-VEGF therapy sensitivity in OV, GBM, LUSC, and CRC, we proposed that AKAP12 might promote resistance via the recruitment and activation of CAFs. Silencing of AKAP12 might decrease the infiltration of tumor-promoting CAFs, thus improving anti-VEGF therapy sensitivity. In addition, a previous study has demonstrated that low AKAP12 methylation levels were associated with its enhanced expression in paclitaxel-resistant OV cells and poor outcomes in OV patients (Bateman et al., 2015). The low methylation status of AKAP12 may also underlie the upregulation of AKAP12 in cancers with anti-VEGF therapy resistance, and predicts worse prognoses. But further studies based on a larger sample of cancer patients are needed to fully explore this hypothesis. Single-cell transcriptomic sequence study implied that AKAP12 expression was predominantly related to metastasis in LUAD. Previous literature convincingly demonstrated that elevated AKAP12 expression in LUAD facilitated cancer cell proliferation, migration, and invasion and suppresses cell apoptosis, a similar study corroborating our conclusion (Chang et al., 2021). This finding jointly confirmed that AKAP12 served as a resistance-related gene in LUAD.

## Conclusion

To sum up, our pan-cancer analysis first revealed that AKAP12 was statistically associated with anti-VEGF inhibitors’ sensitivity, clinical prognosis, DNA methylation, protein phosphorylation, and immune cell infiltration of various cancers, contributing to a more comprehensive appreciation of the role of AKAP12 in tumorigenesis and anti-VEGF resistance and the discovery of possible therapeutic targets.

## Data Availability

The datasets presented in this study can be found in online repositories. The names of the repository/repositories and accession number(s) can be found in the article/Supplementary Material.

## Supplementary material

SUPPLEMENTARY FIGURE S1 The association of INSIG1 expression and sensitivity of anti-VEGF therapy. AKAP12 expression showed upregulation trends in cediranib-resistant NSCLC**(A)** and bevacizumab non-responding CRC patients **(B)**. **(C)** INSIG1 expression was significantly elevated in vandetanib-resistant NSCLC compared to vandetanib-sensitive tissues. INSIG1 expression showed upregulation trends in cediranib-resistant NSCLC **(D)**, bevacizumab-resistant colon cancer **(E)**, and bevacizumab non-responding CRC patients **(F)**. Scatter plot of the association between INSIG1 expression and IC50 values of sorafenib in lung cancer **(C)**, primary pan-cancer **(D)**, and metastasis pan-cancer **(E)**, as well as IC50 values of sunitinib in metastasis pan-cancer **(F)**.

SUPPLEMENTARY FIGURE S2 Prognostic significance of INSIG1 in OV, GBM, CRC patients treated with BV. The prognostic significance of INSIG1 in OV patients **(A)**, GBM patients **(B)**, and CRC patients **(C)** following BV treatment. OS, overall survival; PFS, progression-free survival; PPS, post-progression survival.

SUPPLEMENTARY FIGURE S3 Correlation between AKAP12 expression and OV **(A)** and GC **(B)** patient survival based on Kaplan-Meier database. OS, overall survival; PFS, progression-free survival; PPS, post-progression survival; FP, first progression.

SUPPLEMENTARY FIGURE S4 Expression and protein level of AKAP12 in human tumors**(A)**. The expression level of AKAP12 in TCGA tumors versus adjacent tissues (if available) as shown by TIMER2. **p* < 0.05; ***p* < 0.01; ****p* < 0.001. **(B)** AKAP12 expression in ACC, DLBC, LAML, LGG, MESO, OV, SARC, SKCM, TGCT, THYM, UCS, and UVM (TCGA project) compared with the corresponding normal tissues (GTEx database) based on GEPIA2. **(C)** Total protein levels of AKAP12 in normal tissues and primary breast cancer, colon cancer, HNSC, HCC, LUAD, OV, and UCEC. Protein data were acquired and analyzed by applying CPTAC. **(D)** Comparison of AKAP12 mRNA expression between normal and cancerous tissues and immunohistochemistry images in normal and cancerous tissues. AKAP12 protein expression was significantly lower in BRCA, COAD, UCEC, and OV.

SUPPLEMENTARY FIGURE S5 DNA methylation and protein phosphorylation analysis of AKAP12 in multiple cancers based on the Ualcan database**(A)**. Comparison of the level of AKAP12 methylation between primary tumor tissues versus normal tissues. **(B)** Phosphoprotein sites with a statistical difference in AKAP12 are schematically depicted in a diagram. **(C)** Box plot representation of AKAP12 phosphoprotein levels in BRCA, KIRC, and LUAD.

SUPPLEMENTARY FIGURE S6 The correlation between the AKAP12 expression and infiltration of B cells **(A)**, NK cells **(B)**, CD8+ cells **(C)**, and Treg cells **(D)** among all TCGA tumors according to EPIC, MCPCOUNTER, TIDE, XCELL, TIMER, CIBERSORT, CIBERSORT-ABS as well as QUANTISEQ algorithms.
