# Supplementary figures and images for: Pan-cancer analysis of the prognosis and immunological role of AKAP12: A potential biomarker for resistance to anti-VEGF inhibitors
Source: Front Genet. 2022 Aug 30;13:943006. doi: 10.3389/fgene.2022.943006 (PMC9468827; doi:10.3389/fgene.2022.943006)

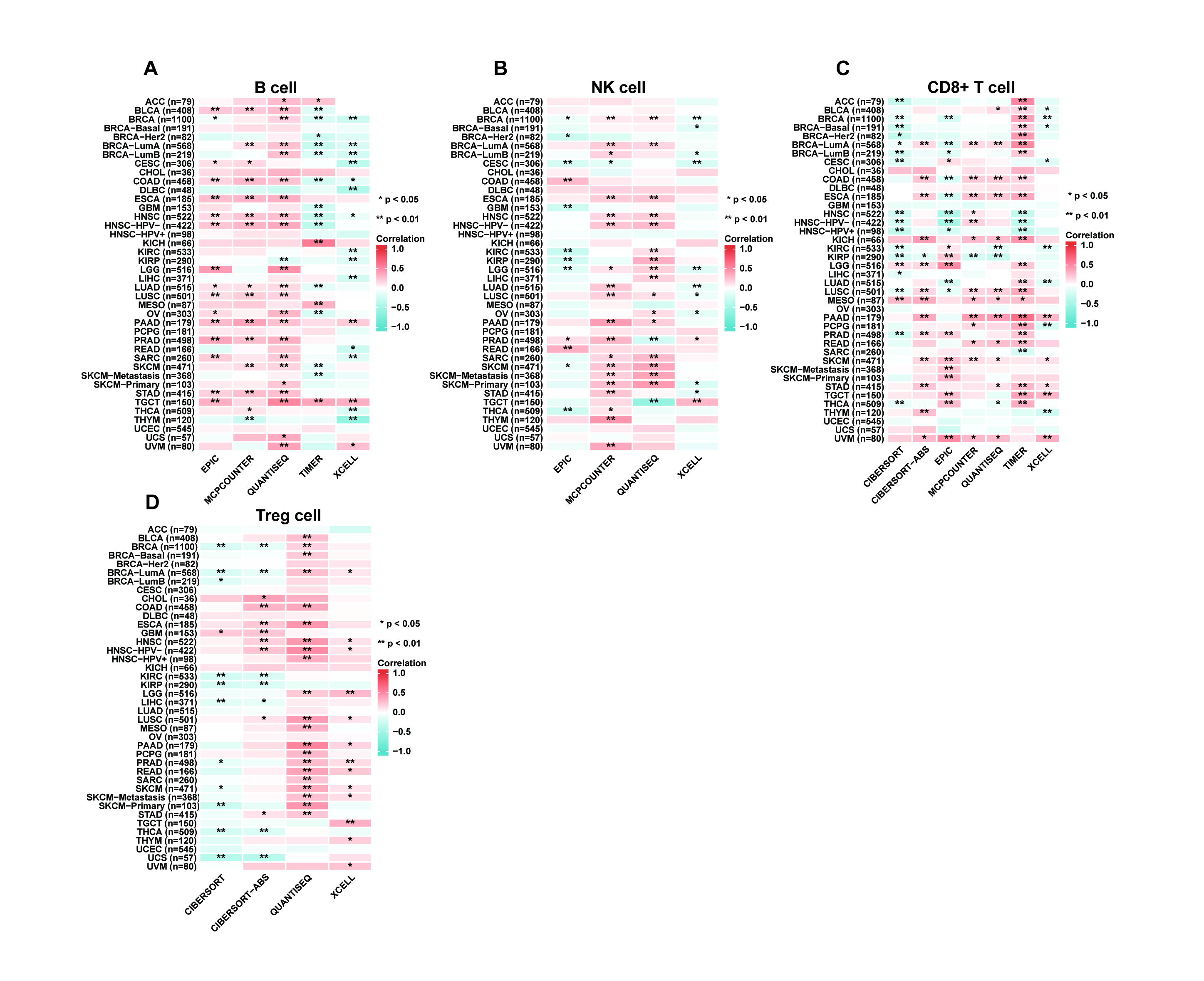

Supplement: Supplementary file 1 [file Image6.tif]

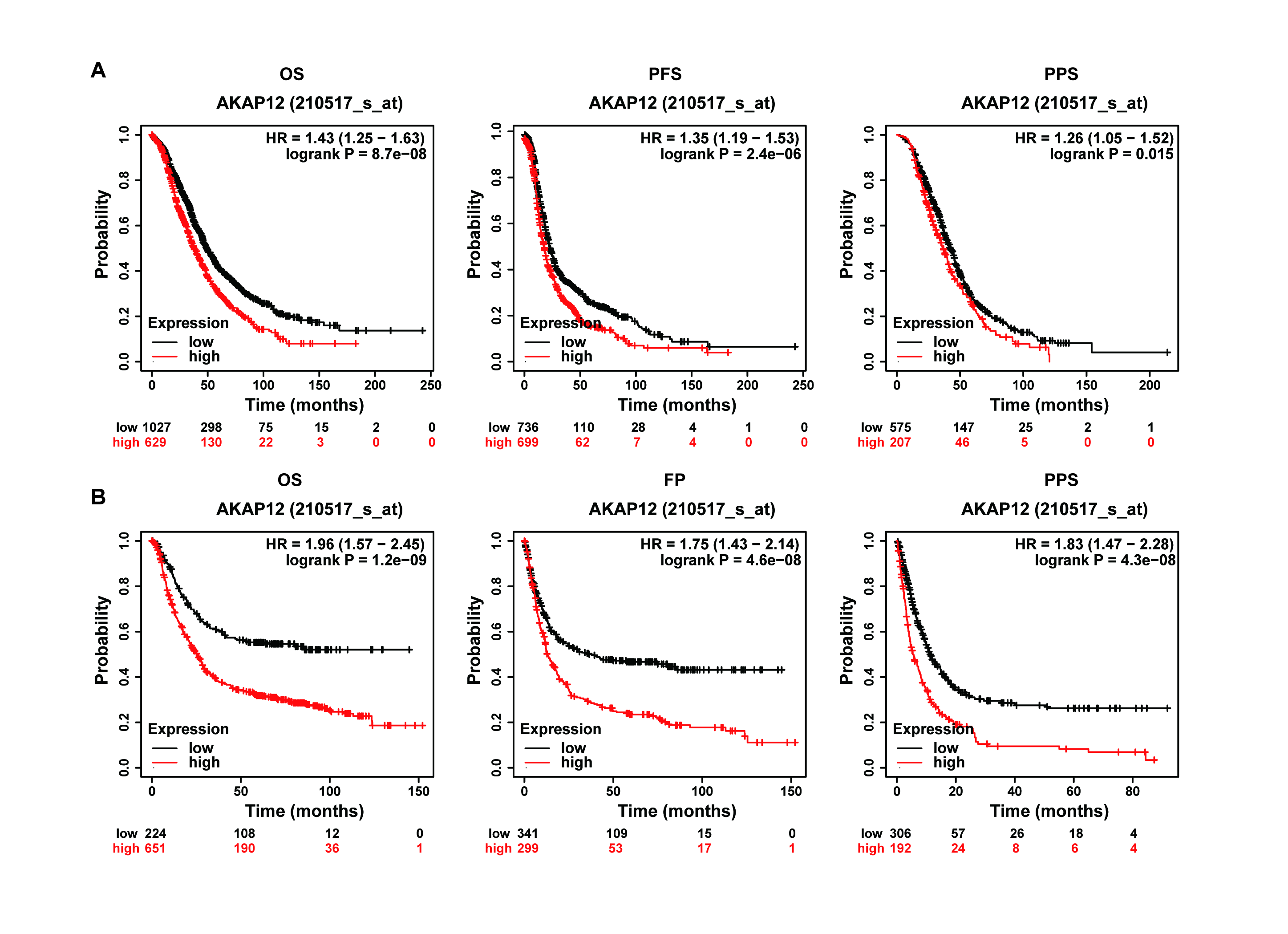

Supplement: Supplementary file 2 [file Image3.tif]

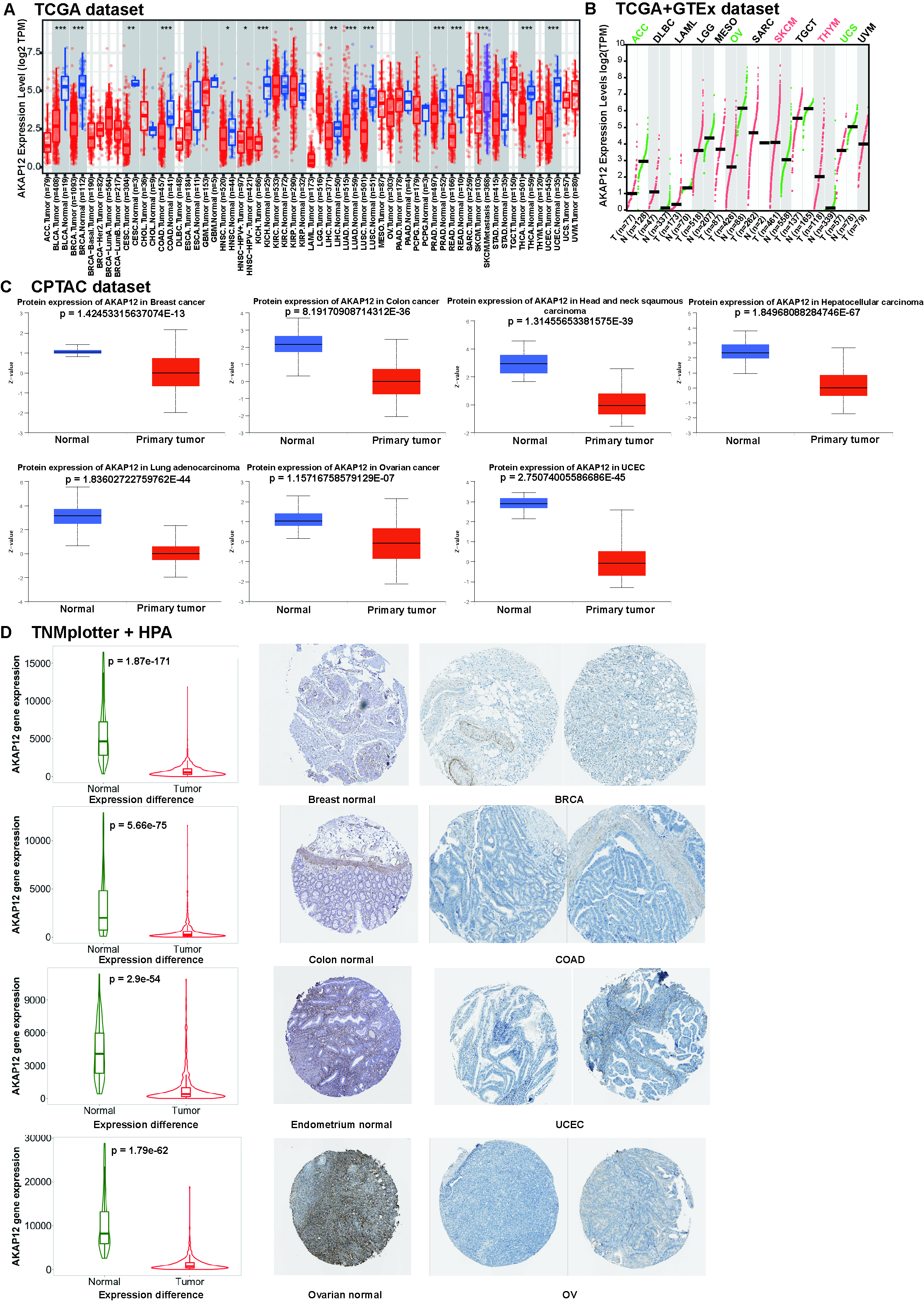

Supplement: Supplementary file 3 [file Image4.tif]

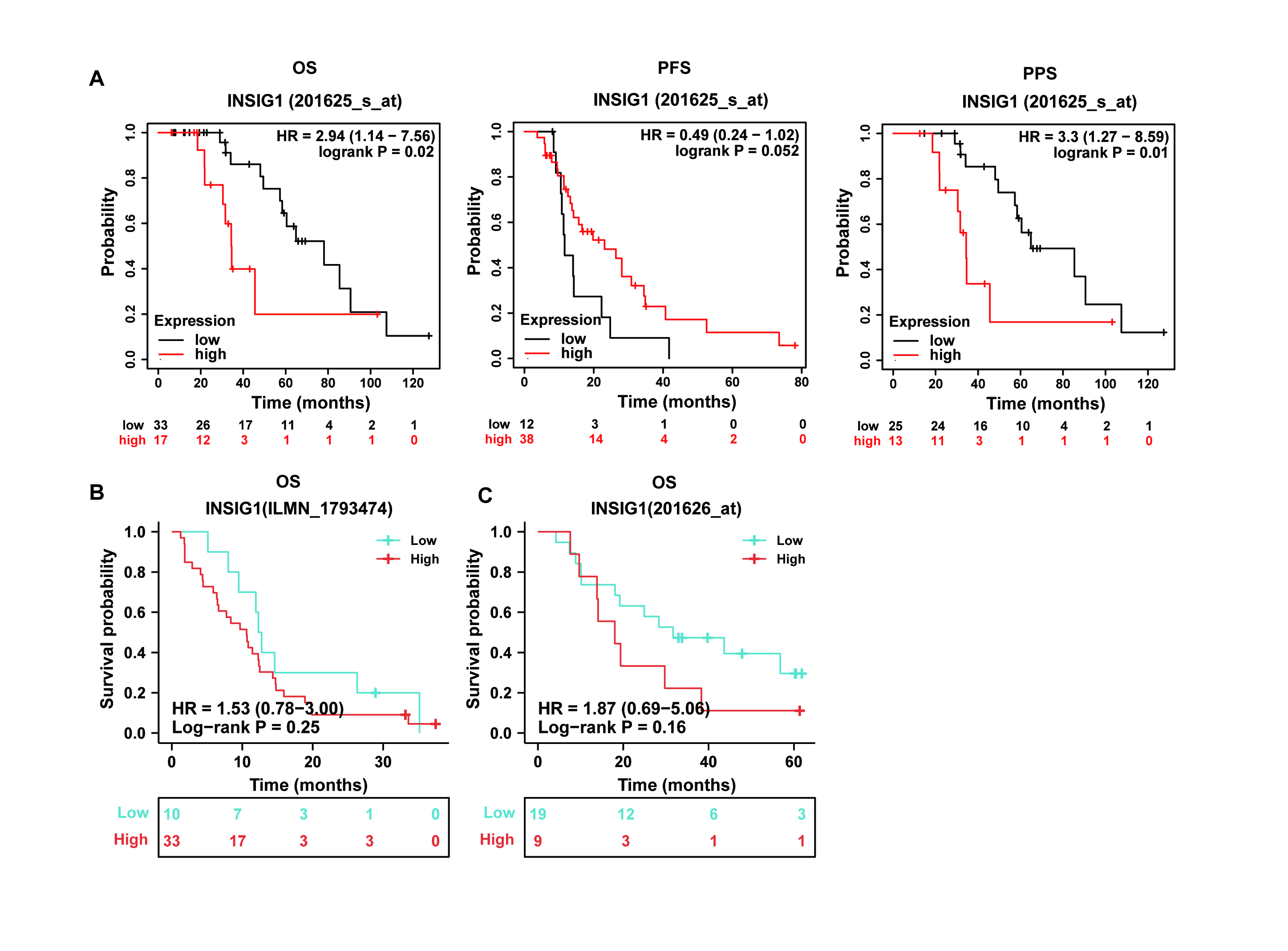

Supplement: Supplementary file 4 [file Image2.tif]

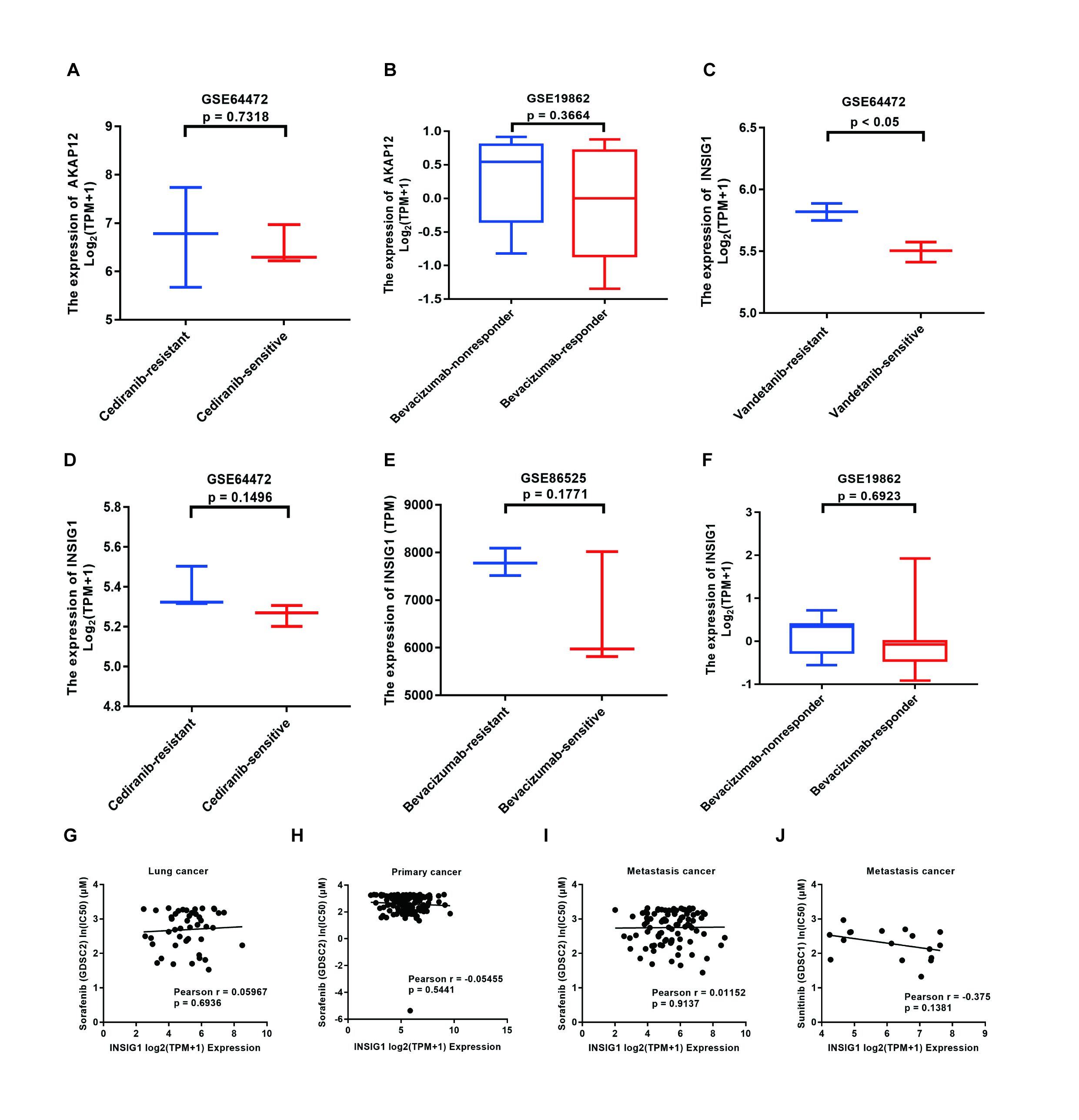

Supplement: Supplementary file 5 [file Image1.TIF]

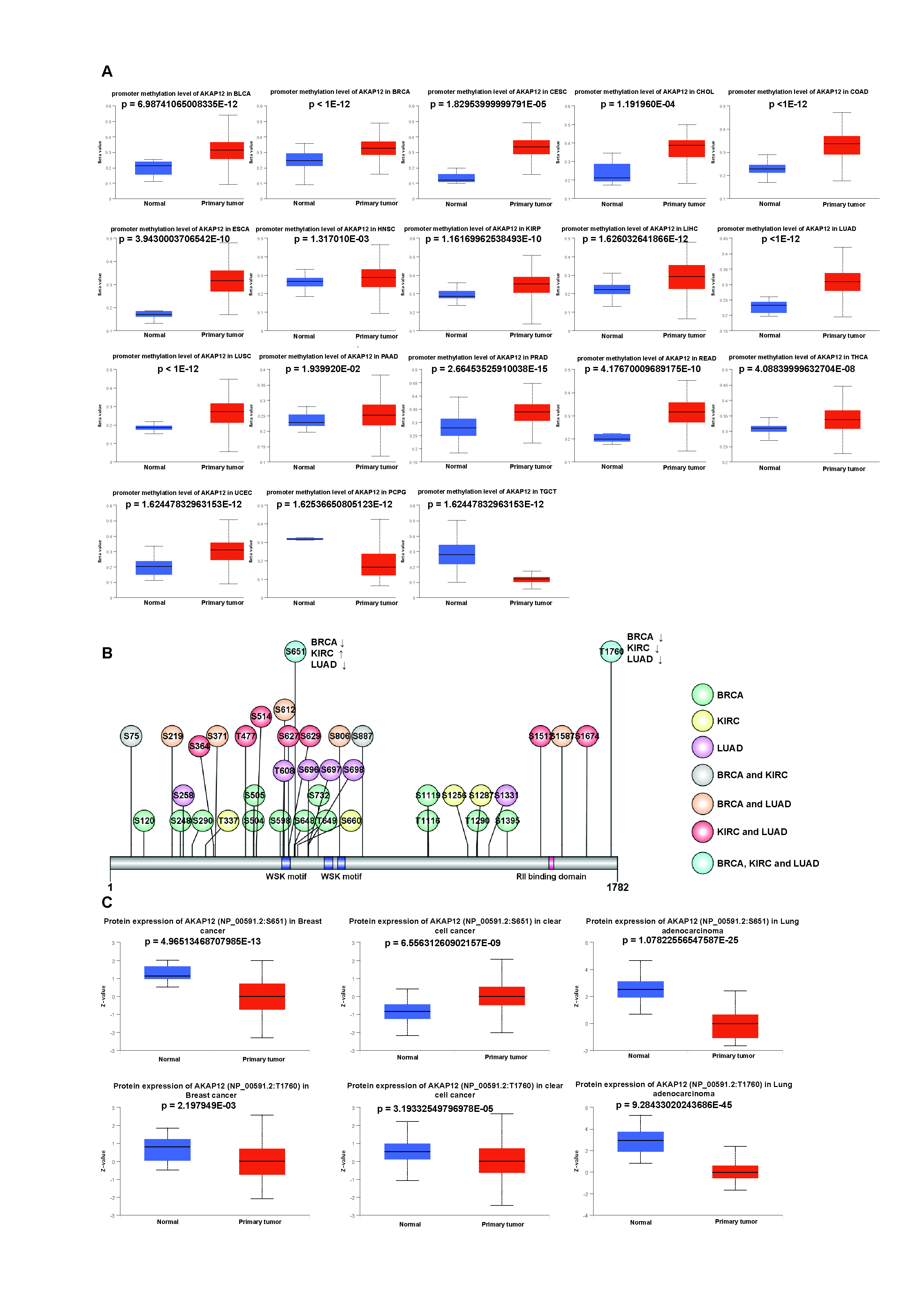

Supplement: Supplementary file 7 [file Image5.tif]
